# Mitochondrial–Immune Overlap in Leber Hereditary Optic Neuropathy: A Case Report and Lessons Learned

**DOI:** 10.3390/reports8040258

**Published:** 2025-12-05

**Authors:** Hind Alnajashi, Walid Eltantawi

**Affiliations:** 1 Department of Neurology, Faculty of Medicine, King Abdulaziz University, Jeddah P.O. Box 80200, Saudi Arabia; 2 Department of Neurology, King Abdulaziz University Hospital, Jeddah P.O. Box 80200, Saudi Arabia; 3 International Medical Centre, Jeddah P.O. Box 80200, Saudi Arabia; drwalidtantawi@gmail.com

**Keywords:** Leber hereditary optic neuropathy (LHON), multiple sclerosis (MS), mitochondrial dysfunction, optic neuritis, transverse myelitis, Harding’s disease

## Abstract

**Background and Clinical Significance**: Leber hereditary optic neuropathy (LHON) is a mitochondrial disorder characterized by acute or subacute bilateral central vision loss, typically in young males. Multiple sclerosis (MS) and neuromyelitis optica spectrum disorder (NMOSD) are immune-mediated demyelinating diseases that may present with optic neuritis and myelitis. Although distinct in etiology, recent evidence suggests that mitochondrial dysfunction and neuroinflammation can overlap, giving rise to combined phenotypes such as LHON-MS (also known as Harding’s disease). **Case Presentation**: We report a 42-year-old man who initially presented in 2018 with right-eye pain and severe visual loss diagnosed as idiopathic optic neuritis. Despite corticosteroid and plasma-exchange therapy, visual recovery was poor, and he was maintained on azathioprine. One year later, he developed visual flashes and left-eye visual loss with bilateral optic nerve thinning on OCT. Genetic testing revealed a pathogenic *MT-ND4* (m.11778G>A) mutation, confirming LHON. In 2021, he presented with ascending lower-limb numbness and bladder urgency. MRI demonstrated a central thoracic cord lesion at T11, consistent with acute transverse myelitis, while serum AQP4 and MOG antibodies were negative. CSF showed five unique oligoclonal bands. The diagnosis of LHON-MS overlap was established, and he was treated with corticosteroids followed by rituximab with clinical stability thereafter. **Conclusions**: This case highlights the diagnostic challenges of LHON with atypical optic neuritis initially followed by the development of demyelinating disease. Red flags such as poor visual recovery, bilateral or sequential optic neuropathy, and steroid-refractory episodes should prompt genetic testing to rule out LHON. Recognition of the mitochondrial–immune overlap is essential for accurate diagnosis, counseling, and an appropriate therapeutic strategy.

## 1. Introduction and Clinical Significance

Leber hereditary optic neuropathy (LHON) is a mitochondrial disorder causing acute or subacute bilateral central vision loss, typically in young males. Multiple sclerosis (MS) and neuromyelitis optica spectrum disorder (NMOSD) are immune-mediated demyelinating diseases that may present with optic neuritis and myelitis. Although pathophysiologically distinct, mounting evidence suggests that mitochondrial dysfunction and neuroinflammation intersect, giving rise to overlapping phenotypes collectively referred to as LHON-MS or Harding’s disease [1,2]. This small but well-described subgroup of patients exhibits both LHON mutations seen in mitochondrial hereditary diseases and inflammatory demyelinating episodes mimicking MS, exceeding coexistence by chance [2]. Published reports describe a heterogeneous group of patients, most of the time diagnosed as MS, and many years later, a retrospective diagnosis of LHON is made [3,4]. Most of these cases are female, with a median age of diagnosis of 30, with painless vision loss [2]. In this case, we present a male patient who presented with painful vision loss suggestive of optic neuritis with poor recovery; a few years later, he had a demyelinating attack with a positive CSF study, making the diagnosis of demyelinating disease on top of the existing LHON diagnosis.

Clinical Significance: This case illustrates the diagnostic dilemma that can be seen in such cases: the patient was initially diagnosed with idiopathic optic neuritis until one year later, when he had involvement in the other eye, warranting genetic testing that confirmed the diagnosis of LHON. Subsequently, this patient was diagnosed with an LHON-MS overlap after developing typical symptoms of demyelinating disease. Increasing awareness of the possible overlaps can improve the diagnostic accuracy, encourage neurologists to consider LHON testing, and improve the treatment pathway.

## 2. Case Presentation

**Figure 1:**
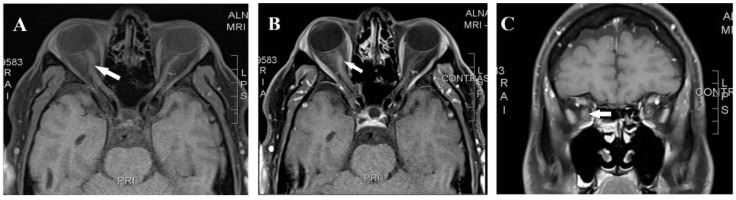
Magnetic resonance imaging axial T1 pre-contrast (**A**), axial T1 post-contrast (**B**), and coronal T1 post-contrast (**C**) showed right-optic-nerve mild enhancement and enlargement (arrow).

A 42-year-old man presented in February 2018 with right-eye pain and progressive visual loss over two weeks. The patient was in his usual state of health with no medical illness other than chronic migraines managed by topiramate. He has no family history of autoimmune disease or neurological disease but mentioned a case of blindness in a maternal uncle, which was attributed to glaucoma. Upon examination, visual acuity was reduced to hand motion OD and 20/20 OS, with a right relative afferent pupillary defect. Fundus examination showed optic disk hyperemia in the right eye; the left eye was normal. Visual evoked potential (VEP) showed an absent right-eye response, and the left-eye P100 = 112 ms. Automated Humphrey visual field testing showed a dense central scotoma in the right eye. Laboratory evaluation and a routine autoimmune panel, like antinuclear antibodies (ANAs), in addition to negative aquaporin-4 antibody (AQP4 Ab) and anti-myelin oligodendrocyte glycoprotein (MOG) antibodies, were negative. Cerebrospinal fluid (CSF) analysis was unremarkable, including negative oligoclonal bands. Brain and spinal MRI were normal, while orbital MRI revealed right-optic-nerve enhancement and mild enlargement (Figure 1). Moreover, metabolic, toxicological, and infectious serological studies were negative. He was treated with intravenous methylprednisolone followed by oral prednisone and plasma exchange with minimal recovery (the right eye improved from hand motion to counting fingers at a 50 cm distance). Azathioprine at a dose of 2 mg/Kg was started for the presumptive diagnosis of idiopathic autoimmune optic neuritis.

One year later, in June 2019, he developed visual flashes and headaches affecting the left eye, with decreased vision. MRI showed no new lesions. Upon examination, vision acuity was 20/200 OD and 20/60 OS with a right relative afferent pupillary defect. Fundus examination showed a pale right optic disk and a subtle temporal pallor left optic disk. VEP demonstrated delayed P100 latency (159 ms) in the left eye and a persistent absent response in the right. Optical coherence tomography (OCT) showed diffuse retinal nerve fiber layer thinning bilaterally. Automated Humphrey visual field testing showed a persistent dense central scotoma in the right eye and a temporal defect in the left eye. The patient received another course of IV methylprednisolone, followed by oral tapering. Due to the development of bilateral optic neuropathy and poor recovery, hereditary causes were favored over inflammatory causes. The patient also underwent an extended workup, including repeated autoimmune antibody testing, thyroid, vitamin B12, and infectious etiology, including bacterial, viral, and syphilis, in addition to the CT chest, abdomen, pelvis, and PET scan, which were all inconclusive. Finally, genetic testing (whole-exome and mitochondrial genome sequencing) revealed a point mutation at the m.11778G>A (MT-ND4) gene, confirming the diagnosis of LHON. The patient was advised to stop azathioprine and referred to the genetics clinic for counseling.

**Figure 2:**
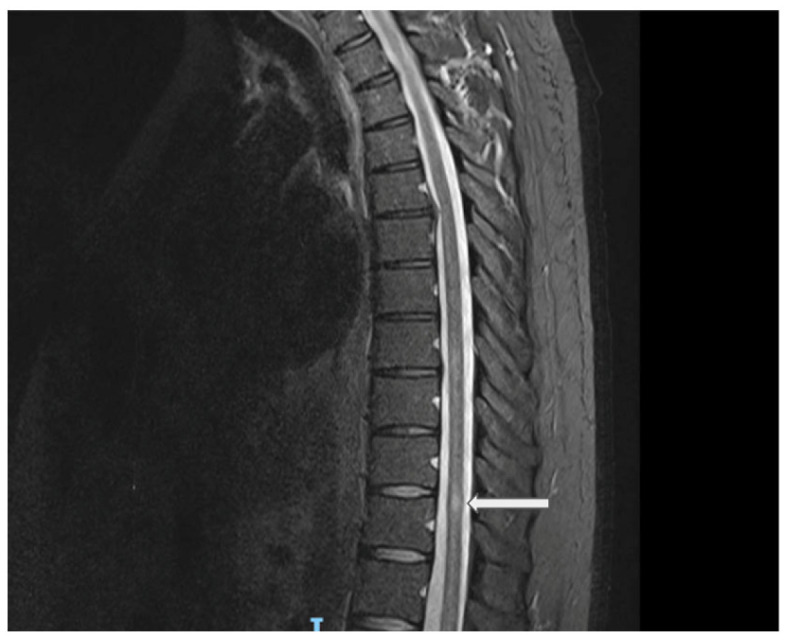
Sagittal spine MRI (STIR sequence) showing a central thoracic cord lesion with high T2 signal intensity opposite the superior aspect of the T11 vertebral level, associated with subtle cord expansion. Explanation of the arrow figure: central thoracic cord lesion with high T2 signal intensity opposite the superior aspect of the T11 vertebral level.

In August 2021, the patient presented with a 10-day history of ascending lower-limb numbness in both legs with some bladder urgency, without weakness or gait disturbance. Neurological examination showed preserved strength and reflexes with sensory impairment to the knees bilaterally; at that time, the estimated expanded disability status scale (EDSS) was 2.5. The brain MRI demonstrated no demyelinating lesions. The spine MRI revealed a small T2-hyperintense lesion at T11 with subtle cord expansion, minimal post-contrast enhancement, and a questionable ill-defined lesion at T8 (Figure 2). These findings were consistent with acute transverse myelitis. Serum AQP4 and MOG antibodies were again negative. CSF was positive for five unique OCBs. An extended diagnostic workup was performed to exclude alternative etiologies of myelitis, including ANA, ENA, ANCA, ACE, B12/MMA, infectious serologies (HIV, VZV, syphilis, TB), and paraneoplastic screening, all of which were unremarkable. He was treated with intravenous corticosteroids with good improvement. After this episode, the patient was diagnosed with LHON plus a demyelinating disease, most likely LHON-MS or Harding’s disease.

The patient was started on rituximab every 6 months as a disease-modifying therapy and was counseled on mitochondrial disease management, including the use of idebenone and vitamin supplementation; also, he was advised to discontinue smoking. The patient’s condition has been stable with no further relapses and some improvement in vision. His last assessment showed a vision of 20/200 OD and 20/20 OS, and he recognized 0/16 color plates in the right eye and 16/16 in the left eye with a pale right optic nerve and mild left-optic-nerve temporal pallor. Otherwise, his neurological exam continues to be the same, with only sensory abnormality estimated at EDSS = 2.

## 3. Discussion

LHON is a mitochondrially inherited disorder due to a point mutation in the mtDNA. LHON is characterized by sequential optic nerve involvement with variable degrees of visual impairment. However, systemic involvement is not uncommon and is referred to as LHON+. The most common organ involvements are the cardiac and nervous system [5]. Several reports mentioned the possible coexistence of mitochondrial disease, LHON, and demyelinating diseases like MS. This coexistence has been given a specific name: Hardig’s disease. The pathophysiology of LHON involves a gene mutation that causes mitochondrial dysfunction, resulting in selective retinal ganglion cell apoptosis [6]. Although this point mutation causes neurodegeneration, it makes patients prone to neuroinflammation and possibly to developing MS [6]. The relation between neuroinflammation and mitochondrial dysfunction remains hypothetical rather than established pathogenic pathways. Evidence from experimental models of multiple sclerosis showed that mitochondrial dysfunction is present even before overt clinical demyelination occurs [7]. Similarly, several studies reviewed the potential role of mitochondrial dysfunction in MS, highlighting that mitochondrial respiratory chain deficiencies and altered mitochondrial dynamics may contribute to neurodegeneration in MS, although the direct causation of these associations remains incompletely understood [8]. While these observations offer biologically plausible explanations for cases of LHON–MS overlap, a definitive link is still under investigation, and current evidence should be interpreted as supportive but not conclusive. A review of 103 cases with LHON found that three mutations (m.11778G>A, m.3460A>G, and m.14484T>C) are associated with demyelinating symptoms like MS. They reported that MS symptoms in these patients are almost similar to any patient with MS, with only a more severe visual involvement [1]. Our patient presented initially with unilateral severe optic neuritis, manifesting as marked vision loss, eye pain, subacute onset, and abnormal MRI features. These features initially deviated the working diagnosis from a hereditary neurodegenerative etiology. An MRI of the brain in isolated LHON is expected to be normal or can show some optic nerve atrophy at later stages [9]. However, the description of optic nerve hyperintensity, especially retro-chiasma and chiasm enlargement and T2 signals in MRI, which can mimic optic neuritis, has been described in few cases of LHON [10,11]. These MRI findings can cause diagnosis complexity and expose the patient to multiple immunotherapy treatments; the lack of visual improvement despite the use of immunotherapy like steroids or plasma exchange should prompt considering a genetic test for LHON. A previous report by Lai and colleagues addressed the main similarities and differences in clinical features between two groups of LHON and optic neuritis Chinese patients during the acute stage [12]. Evaluation of clinical features of both LHON and inflammatory optic neuritis could clarify the overlap between both conditions, especially in the acute stage (Table 1) [12,13,14].

To illustrate the diagnostic complexity of our case, we reviewed published reports describing LHON–MS overlap (Harding’s disease). Table 2 summarizes representative cases and highlights how phenotypic variability, diagnostic delay, and overlapping MRI/CSF features may complicate accurate classification. These comparisons underscore the distinctive features of our patient, particularly the confirmation of a pathogenic LHON mutation prior to the onset of myelitis and the presence of optic nerve enhancement—an uncommon but increasingly recognized challenge in differentiating LHON from immune-mediated optic neuritis.

Patients with LHON-MS and patients with MS share almost similar features on brain MRI, fulfilling the 2017 McDonald’s diagnostic criteria for MS [17]. Our case had only a spinal cord lesion and no other lesions to fulfill dissemination in space (DIS) criteria (requiring at least one lesion in two topographical locations typical for MS) and had a positive oligoclonal band in the CSF study at the time of the transverse myelitis presentation, which could substitute for dissemination in time [12]. A more recently published diagnostic criterion for MS, the 2024 revisions of the McDonald Criteria, added the presence of an optic nerve lesion on MRI as a fifth topographical lesion for dissemination in space (DIS) [18]. So our case can be diagnosed as MS after excluding any alternative diagnosis according to the newer version of the 2024 McDonald Criteria, based on the presence of two lesions (optic nerve and spinal cord) and a positive CSF study. Nevertheless, applying the 2024 McDonald Criteria in this context is challenging. The patient carries a confirmed mitochondrial mutation causing hereditary optic neuropathy, providing a clear alternative explanation for optic nerve pathology. Thus, complete exclusion of a non-MS etiology for optic nerve involvement—one of the essential prerequisites when applying MS diagnostic criteria—cannot be definitively achieved in this case. This overlap between hereditary mitochondrial optic neuropathy and inflammatory spinal cord disease illustrates the diagnostic ambiguity characteristic of LHON-MS overlap syndromes (Harding’s disease). It highlights the need to interpret the 2024 McDonald Criteria cautiously, especially in patients with atypical presentations, to avoid misdiagnosis.

There is not enough evidence to support specific treatment options for Harding’s disease or LHON-MS. Disease-modifying therapy used in MS can be used in addition to a specific treatment for LHON, like a trial of idebenone. Until now, there has been no cure for LHON; idebenone showed some benefit in stabilizing vision, but results have been inconsistent [19].

LHON is a challenging diagnosis, as the initial presentation can mimic multiple sclerosis or other autoimmune demyelinating diseases, like NMOSD or myelin oligodendrocyte antibody-associated disease (MOGAD). In order to reach the diagnosis, an extensive workup to exclude disease mimics and genetic testing is required. Red flags in patients with MS, for example, the presence of severe optic nerve dysfunction or subsequent optic nerve improvements in a patient with MS, may sometimes warrant testing for LHON, as this disease requires a specific approach and genetic counseling. Although LHON–MS overlap syndromes have been previously reported, the sequence observed in our patient is unusual and clinically important. In most published cases, patients are diagnosed with MS (or recurrent optic neuritis) first, and LHON is only recognized later when vision fails to recover. In contrast, our patient had genetically confirmed LHON prior to the development of a typical demyelinating event, which provides a unique window into how mitochondrial neurodegeneration may precede or coexist with central nervous system demyelination.

This case also reinforces the importance of considering LHON in patients with atypical optic neuropathy, particularly when visual recovery is poor, when MRI findings mimic inflammatory disease, or when treatment response is minimal. While ophthalmologists frequently encounter LHON-like presentations, neurologists may be less familiar with the condition, increasing the risk of misclassification as inflammatory optic neuritis or CRION. Increased awareness of LHON testing among neurologists is therefore essential to avoid diagnostic delay and to appropriately interpret subsequent demyelinating events.

## 4. Conclusions

This case illustrates the importance of recognizing that LHON can present in the context of inflammatory demyelinating disease, resulting in a diagnostic overlap known as LHON-MS or Harding’s disease. The initial presentation may resemble autoimmune optic neuritis. However, red flags, such as poor visual recovery, sequential optic nerve involvement, and no improvement with steroids, should prompt consideration of genetic testing for LHON, even in patients who appear to have demyelinating features. Although this case does not present a new or novel diagnosis, it illustrates and reinforces the diagnostic challenges of distinguishing LHON from inflammatory optic neuritis.

What is also unique in this case is the sequence of diagnosis. LHON was genetically confirmed before the patient developed typical inflammatory myelitis, underscoring the need to consider LHON in atypical or poorly recovering optic neuritis. Furthermore, the subsequent development of myelitis and positive CSF oligoclonal bands in this patient describes how mitochondrial dysfunction may predispose to immune-mediated neuroinflammation. Identification of LHON-MS overlap is critical, as it has implications for therapeutic decisions, guides genetic counseling, and helps to avoid unnecessary immunosuppression in cases where inflammation is not the main cause of vision loss.

## Figures and Tables

**Table 1:** Features of typical idiopathic optic neuritis vs. Leber’s hereditary optic neuropathy (LHON) in adults.

	Idiopathic Optic Neuritis	Leber’s Hereditary Optic Neuropathy
Age of onset	Typically <45 years, but may be of any age	The usual age is between 15 and 35 years
Gender	Female preponderance about (75%)	Male preponderance (80–90%)
Family history	Not common	Common (maternally inherited)
Onset	Commonly acute (within days)	Commonly subacute (within weeks)
Bilateral ON	Commonly unilateral	Bilateral sequential 75% or simultaneous 25%
Pain	Periocular pain (90%), with movement	Commonly painless
Visual acuity	Unilateral loss—variable severity	Bilateral loss—severe before stabilization
Optic disk	Normal (65%) or swollen (35%)	Normal (30%) or hyperemic swelling (70%)
RAPD	Commonly ipsilateral to affected eye	Usually not present
Imaging	Abnormal optic nerve enhancement in 90%	Normal orbital imaging in >90%
Pathology	Demyelination of optic nerve	mtDNA mutation with impaired ATP synthesis
Treatment	IV methylprednisolone (anti-inflammatory)	Idebenone (mitochondrial neuroprotection)
Prognosis	Recover in >90% within weeks	Worsen over months then stabilize at 95%

**Table 2:** Published LHON–MS overlap cases compared with the present case.

Study/Reference	Age/Sex	LHON Mutation	Initial Optic Neuropathy Features	MRI Brain/Optic Nerve Findings	CSF (OCB/IgG Index)	Treatment Used	Key Points/How the Present Case Differs
Chang et al. (BMJ Case Rep) [15]	28/M	m.11778G>A	Sequential visual loss over 8 months	Brain MRI with typical MS lesions	OCB positive	Glatiramer acetate	Brain MRI fulfilled MS criteria early, unlike our case, which had a normal brain MRI initially
Holmøy et al. (BMC Neurol) [3]	24/F	m.11778G>A	Sequential visual loss	Brain MRI with typical MS lesions	OCB positive	Interferon-β, natalizumab	MS diagnosed long before LHON
Joshi & Kermode (BMJ Case Rep) [4]	20s/F	m.11778 mtDNA	Sequential visual loss 15 years apart	Brain MRI with typical MS lesions	Not performed	None reported	Diagnosis of LHON delayed by decades
Riccio et al. (Can J Ophthalmol) [16]	30/F	m.11778G>A	Sequential visual loss	Brain MRI with typical MS lesions	Not reported	Glatiramer acetate	MS family history; LHON identified after recurrent ON
Present Case	42/M	m.11778G>A (MT-ND4)	Painful acute ON sequential involvement over 1 year	Right-optic-nerve enhancement, no brain MS lesions; later thoracic T11 myelitis; normal brain MRI	Initial CSF normal; later OCBs positive	Rituximab, idebenone	Unique sequence: LHON genetically confirmed before demyelinating event; MRI enhancement atypical for LHON

## Data Availability

The original contributions presented in this study are included in the article. Further inquiries can be directed to the corresponding author.

## Abbreviations

The following abbreviations are used in this manuscript:

Abbreviation	Definition
LHON	Leber hereditary optic neuropathy
MS	Multiple sclerosis
NMOSD	Neuromyelitis optica spectrum disorder
LHON-MS	LHON associated with demyelinating disease (Harding’s disease)
MT-ND4	Mitochondrial NADH dehydrogenase subunit 4 gene
MRI	Magnetic resonance imaging
STIR	Short tau inversion recovery
OCT	Optical coherence tomography
RNFL	Retinal nerve fiber layer
GCL	Ganglion cell layer
VEP	Visual evoked potential
RAPD	Relative afferent pupillary defect
CSF	Cerebrospinal fluid
OCBs	Oligoclonal bands
AQP4 Ab	Aquaporin-4 antibody
MOG Ab	Myelin oligodendrocyte glycoprotein antibody
ANA	Antinuclear antibody
ENA	Extractable nuclear antigen
ANCA	Anti-neutrophil cytoplasmic antibody
IVMP	Intravenous methylprednisolone
AZA	Azathioprine
DMT	Disease-modifying therapy
DIS	Dissemination in space (MS diagnostic criterion)
DIT	Dissemination in time (MS diagnostic criterion)
